# Assessment of quality of life using WHOQOL-BREF in patients with visceral leishmaniasis

**DOI:** 10.1186/s12955-019-1112-2

**Published:** 2019-03-28

**Authors:** Rajendra Babu Veeri, Ashok Kumar Gupta, Biplab Pal, Niyamat Ali Siddiqui, Devi Priya, Pradeep Das, Krishna Pandey

**Affiliations:** 1 Department of Pharmacy Practice, National Institute of Pharmaceutical Education and Research (NIPER), Hajipur, 844102 India; 2 Department of Bio Statistics, Rajendra Memorial Research Institute of Medical Sciences (RMRIMS), Agamkuan, Patna, 800007 India; 3 Department of Molecular Biology, Rajendra Memorial Research Institute of Medical Sciences (RMRIMS), Agamkuan, Patna, 800007 India; 4 Department of Clinical Medicine, Rajendra Memorial Research Institute of Medical Sciences (RMRIMS), Agamkuan, Patna, 800007 India

**Keywords:** Quality of life, Visceral leishmaniasis, WHOQOL-BREF

## Abstract

**Background:**

This study was aimed to assess the impact of quality of life using WHOQOL-BREF in patients with Visceral leishmaniasis (VL).

**Methods:**

A total of 95 VL cases and 95 healthy participants filled out the questionnaires. Data on socio-demographic aspects along with disease duration were collected. Data were compared using a t-test, analysis of variance and chi-square test.

**Results:**

VL patients experienced very high impact on their quality of life. Study cohort had male preponderance (72.63%). Majority (64.21%) were aged < 40 years. Longer disease duration was found to have significantly poor quality of life (*p* < 0.05). The physical domain was found to be most affected domains of quality of life (QOL). QOL was affected most in illiterate, married, housewife, rural population and patients with longer disease duration (*p* < 0.05). The psychological and environmental domains were significantly affected in > 40 years of age group married patients (*p* < 0.05).

**Conclusions:**

VL significantly impaired the patients’ (QOL) in all four domains (physical, psychological, social relationship and environmental). Physical domain was significantly the most affected domain.

## Background

Visceral leishmaniasis (VL) or Kala-azar is a major health issue in many regions across the world [1]. VL is a systemic infection caused by obligate intracellular protozoal parasite *Leishmania donovani* [2]. The disease is transmitted through the bite of infected female phlebotomine sandflies [3]. VL is fatal if left untreated [4] with presenting features include fever, often associated with rigor and chills, abdominal discomfort, splenomegaly, pancytopenia, weight loss and weakness. Secondary infections such as tuberculosis (TB), human immune-deficiency virus (HIV) and hemorrhage are the leading causes of mortality. VL is the second leading cause of death by intracellular parasite next after malaria [5] and affects poorest of poor people living in rural areas. The disease has spreads across South America, Africa, Mediterranean countries and southeast Asian countries, including China, Bangladesh, Nepal and India [6]. In India, the disease is prevalent in most of North-Eastern states like Bihar, Jharkhand, West Bengal, Assam and Uttar Pradesh. Nearly 50% of the world’s Kala-azar is reported from Bihar region [7].

An estimated 700,000 to 1 million new cases and 20,000 to 30,000 deaths occur annually worldwide [8]. Access to proper diagnosis and treatment in this group of population is critical. Limited treatment choices are available for the management of VL with significant pharmacoeconomic burden. Due to lack of sensitivity sodium antimony gluconate (SAG) is no more in use in Bihar region [9]. Other treatment options such as Miltefosine, Paromomycin and Amphotericin B are often associated with many side effects so their widespread usage are limited [10–12]. However, liposomal formulation of amphotericin B has longer half-life, less toxic [13] and recommended by WHO for elimination of VL from the Indian subcontinent.

QOL is defined by the World Health Organization (WHO) as “an individual’s perception of their position in life in the context of the culture and value systems in which they live and in relation to their goals, expectations, standards and concerns” [14]. Beside these epidemiological findings and intention towards patient care management based on reduction of sign and symptoms and prevention of mortality, QOL assessment has capability to explore physical, social and psychological impact of the disease. Measurements of health related quality of life, particularly in chronic diseases are essential [15]. VL patient, in addition to common clinical symptoms has, to deal with several physical, social, psychological and environmental problems [16]. Also, the treatment itself associated with several side effects. All issues related to disease and its management certainly have a huge impact on the overall well-being of the VL patient. Despite high endemicity of the VL in India and many other parts of the world, to the best of our knowledge no study was done on QOL in VL on this group of patients. QOL studies on various chronic disorders such as cutaneous leishmaniasis (CL) [17], HIV-VL co-infection [18], dengue [19], malaria [20] and filariasis [21] have been reported by using WHOQOL questionnaire. In this study we assessed the impact of VL on health related quality of life by using World Health Organization Quality of Life Instrument, WHOQOL-BREF questionnaire.

## Methods

### Study setting and study design

This was a questionnaire based single center, cross-sectional study carried out from January 2017 to November 2017 at Rajendra Memorial Research Institute of Medical sciences (RMRIMS), Indian Council of Medical Research (ICMR), Patna, Bihar, India. The RMRIMS is the sole referral center for the diagnosis and treatment of VL in the state of Bihar. Patients with signs and symptoms of VL attending the outpatients department of RMRIMS were transferred to an indoor ward for confirming diagnosis and treatment. Patients of VL were admitted in the indoor ward were enrolled in the study.

### Participants

A total of 116 patients were admitted in the inpatient department of RMRIMS during the study period, of which 7 patients did not agree to take part in the study and 14 patients were excluded due to excessively missing data. Therefore, a total of 95 patients were included in the present study. Inclusion criteria consisted of newly diagnosed VL cases with positive rK39 test, further confirmed by microscopically patent parasites in bone marrow/splenic aspirates of either gender, aged ≥18 years. The subjects comprised of 69 males, 26 females, aged between 18 to 66 years. Concurrent febrile etiologies as indicated in differential diagnosis were precluded by appropriate laboratory investigations by the attending clinicians. Patients with HIV, Hepatitis B and C, tuberculosis and any other chronic illness were excluded. All the patients were treated with single dose Ambisome at 10 mg/ kilogram body weight. Patient’s relatives/friends (≥18 years) accompanying with them during hospitalization, who were apparently healthy by physical examination, included in the control group. Control group comprised of 66 men and 29 women, aged between 18 to 62 years. An attempt was done to match case and control group with regards to age, gender, marital and socio economic status.

### Ethical assertion

This study was approved by the Institutional Ethical Committee of Rajendra Memorial Research Institute of Medical Sciences (RMRIMS), Patna (Approval No.06/RMRI/EC/2017). Questionnaire was administered only after the purpose of the study had been clearly explained to the participants and a written informed consent in Hindi was signed. Patients were assured about the anonymity and confidentiality of data.

### Data collection

The questionnaires were filled twice i.e. before initiation of treatment and after 1 month of treatment completion by all the patients (both literates and illiterates). Literate patients have filled the questionnaire by themselves after they had received the required instructions, whereas for illiterate patients, a face-to-face interview was conducted and control group have filled the questionnaires themselves only once.

The WHOQOL-BREF questionnaire is available in multiple languages, and in the present study Hindi version of such questionnaire was used to assess the QOL [22]. WHOQOL–BREF Hindi questionnaire was used to assess the QOL [23]. It consists of 26 questions, of which 24 were divided in four domains, physical, psychological, social relations and environmental and remaining two questions should have self-perceived QOL and satisfaction with health. Each domain is represented by several facets and questions are formulated for a Likert response scale, with intensity (nothing - extremely), capacity (nothing - completely), frequency (never - always) and assessment scales (very dissatisfied - very satisfied; very bad - very good), all of them consisting of five levels (one to five). The obtained raw score was converted to transformed score by using SPSS syntax, which directly converts the raw score into the transformed domains score (the scores are transformed on a scale from 0 to 100 to enable comparisons to be made between domains composed of unequal numbers of items) [24]. Five-level scores are more advisable because of their capacity to measure extremes as well as intermediary accessibility scores [25].

### Data analysis

Continuous variables were summarized as mean with standard deviation (SD) and categorical variables were summarized as frequency with proportion. Socio-demographic variables were compared in between case and control groups using Pearson’s chi-square or Fisher’s exact test. Mean QOL scores across the domains were compared using independent t-test. Association of socio-demographic variables with WHOQOL-BREF domains were determined using independent t-test or one way ANOVA. All tests of significance were two-tailed with a *p*-value < 0.05 indicating a statistically significant difference. Data analyses were performed using the Statistical Package for Social Sciences (SPSS v16.0).

## Results

Total 95 VL patients and 95 healthy controls were recruited in the study. The Majority of patients (72.6%) were males and belong to 18–40 years age group, were illiterate and live in rural areas. The details of socio-demographic characteristics of the VL patients and the control group are shown in Table 1. Higher reporting of male patients (72.6%) in comparison to females, as well as comparatively younger age (64.2%) population was more affected with VL. As for marital status, suffering of married (77.8%) population was observed more. The number of illiterate patients (50.5%) affected with VL found higher with other educational classes. Regarding occupation, labor workers (65.2%) and patient who belongs to rural areas (87.3%) have higher numbers (Table 1).

**Table 1:** Comparison of socio-demographic profile between patients and healthy controls

Variables	Patients N (%)	Control N (%)	*p*-value
Gender			
Male	69 (72.6%)	66 (69.4%)	0.749
Female	26 (27.3%)	29 (30.5%)	
Age (Years)			
18–40	61 (64.2%)	64 (67.3%)	0.760
41–66	34 (35.7%)	31 (32.6%)	
Marital status			
Single	21 (22.1%)	25 (26.3%)	0.612
Married	74 (77.8%)	70 (73.6%)	
Education			
Illiterate	48 (50.5%)	41 (43.1%)	0.413#
Primary school	19 (20.0%)	23 (24.2%)	
Secondary school	13 (13.6%)	12 (12.6%)	
Tertiary	15 (15.7%)	19 (20.0%)	
Occupation			
Labor	62 (65.2%)	56 (58.9%)	0.424#
Housewife	15 (15.7%)	17 (17.8%)	
Others	18 (18.9%)	22 (23.1%)	
Locality			
Urban	12 (12.6%)	18 (18.9%)	0.320
Rural	83 (87.3%)	77 (81.0%)	

The proportions were compared using chi-square or Fisher’s exact test

*p*-value< 0.05 indicating a statistically significant difference

#Chi-square linear-by-linear association

Table 2 shows the means, SDs (Standard deviations) and raw scores for each item of WHOQOL-BREF. The Q1 and Q2 are overall QOL and general health questions and reaming questions were included in four domains. Three items (Q3, Q4 and Q26) were reverse scoring items. The highest and lowest mean scores were observed in before treatment patients’ group were safety (3.60) and physical pain (2.29), respectively.

**Table 2:** Scores of WHOQOL-BREF items among the before and after treatment of VL patients and control group

WHOQOL-BREF Items/Domains	Direction of Scaling	Mean Raw Item Score	Standard Deviation (SD)	Mean Raw Item Score	Standard Deviation (SD)	Mean Raw Item Score	Standard Deviation (SD)
		Before Treatment (*n* = 95)		After Treatment (*n* = 95)		Control group (n = 95)	
Q1 Overall QOL	Positive	1.97	0.69	2.98	0.76	3.97	0.74
Q2 General health	positive	1.97	0.74	2.97	0.71	4.09	0.62
Domain 1: Physical Health							
Q3 Physical pain	negative	2.29	0.68	3.54	0.63	2.88	1.15
Q4 Dependence medication	negative	2.45	0.60	3.03	0.91	2.66	0.98
Q10 Energy	positive	2.89	0.78	2.95	0.76	3.58	0.75
Q15 Mobility	positive	2.55	0.67	2.91	0.67	3.56	0.95
Q16 Sleep and rest	positive	2.57	0.68	3.06	0.82	4.16	0.73
Q17 Activities of daily living	positive	2.74	0.67	2.81	0.76	3.69	0.84
Q18 Working capacity	positive	2.63	0.79	2.92	0.75	3.96	0.73
Domain 2: Psychological Health							
Q5 Life enjoyment	positive	2.79	0.72	2.99	0.87	4.18	0.79
Q6 Meaningfulness of life	positive	2.80	0.75	3.09	0.80	3.53	0.88
Q7 Concentration	positive	2.67	0.70	2.98	0.81	3.24	0.87
Q11 Body appearance	positive	3.0	0.76	3.08	0.75	4.15	0.77
Q19 Self-esteem	positive	2.96	0.68	3.18	0.74	3.93	0.73
Q26 Negative feelings	negative	2.89	0.86	3.16	0.76	4.26	0.70
Domain 3: Social Relationships							
Q20 Personal relationship	positive	3.15	0.81	3.28	0.81	4.07	0.67
Q21 Sexual activity	positive	2.73	0.98	3.84	0.54	4.52	0.56
Q22 Social support	positive	3.39	0.76	3.42	0.77	3.74	0.92
Domain 4: Environment							
Q8 Safety	positive	3.60	0.81	2.95	0.82	4.05	0.77
Q9 Physical environment	positive	3.52	0.86	2.83	0.81	3.01	1.01
Q12 Financial resources	positive	2.69	0.91	2.89	0.69	3.02	0.97
Q13 Daily information	positive	2.44	0.75	2.86	0.82	3.44	0.86
Q14 Leisure	positive	2.41	0.68	2.88	0.76	3.08	0.89
Q23 Home environment	positive	2.56	0.74	3.00	0.74	2.64	0.99
Q24 Access to health care	positive	2.38	0.67	2.89	0.68	3.47	0.96
Q25 Transport	positive	2.55	0.69	2.86	0.68	3.62	0.83

Data are presented as mean and standard deviation (SD)

In Table 3, the comparison between VL patients and control subjects reflects a highly significant affection on QOL observed in all domains of the WHOQOL-BREF. In all four domains the highest and lowest score belongs to social relationship and physical domain, respectively.

**Table 3:** Mean Quality of Life scores in different domains among disease and healthy control

Domains	Before treatment(BT)	After treatment(AT)	Control	*P* Value	
	Mean ± SD			BT Vs AT	BT Vs Control
Physical	39.74 ± 7.0	46.69 ± 9.44	65.37 ± 10.0	0.001	0.000
Psychological	46.32 ± 8.37	50.70 ± 10.69	61.49 ± 9.25	0.002	0.000
Social	60.52 ± 14.09	62.54 ± 13.66	75.65 ± 12.0	0.318	0.000
Environmental	44.21 ± 8.06	47.43 ± 10.97	57.33 ± 8.78	0.022	0.000

The values are represented in mean and standard deviation (SD)

Independent sample t-test was used for comparison

*p*-value< 0.05 indicating a statistically significant difference

*p*-value< 0.001 indicating a statistically highly significant difference

All the patients were treated with single dose Ambisome at 10 mg/ kilogram body weight. After 1 month of receiving treatment we noticed that physical, psychological and environmental domains of QOL score were significantly improved. The maximum score which reflects better QOL were obtained in social relationship domain and minimum score indicates poor QOL obtained in physical domains, respectively.

The mean score of four domains and the total score of the WHOQOL-BREF according to the socio-demographic parameters are depicted in Table 4. There is no gender differentiation with respective to QOL in this study and the QOL of older age (> 40 years) patients were observed with significant (*p* < 0.05) deterioration in their overall QOL. Significantly poor QOL was observed in married persons with regard to all domains (*p* < 0.05) except social domain. With regards to education, illiterate patients observed poor QOL in physical domain when compared to educated patients (*p* < 0.05). Similarly laborers’ displayed lower QOL than patients involved with other occupation in physical and overall domains. Patients living in rural areas, observed with statistically significant worsening with respect to domain 1 (*p* < 0.05) in comparison to urban area patients. Patients’ having longer duration of disease significantly causes more deterioration in physical domain (*p* < 0.05) and overall QOL (*p* < 0.05).

**Table 4:** Association of socio-demographic variables with WHOQOL-BREF domains

Variables	Domain-1	Domain-2	Domain-3	Domain-4	Overall QOL
Gender					
Male	39.44 ± 6.89	45.47 ± 7.82	59.72 ± 14.01	44.34 ± 8.48	47.24 ± 5.98
Female	40.52 ± 6.22	48.56 ± 9.50	62.66 ± 14.36	43.87 ± 6.99	48.90 ± 6.20
*p*-value	0.467	0.147	0.376	0.785	0.247
Age (In Yrs)					
18–40	40.52 ± 6.68	47.68 ± 8.00	61.54 ± 12.91	45.65 ± 7.87	48.85 ± 5.55
41–66	38.34 ± 6.60	43.87 ± 8.60	58.70 ± 16.03	41.64 ± 7.87	45.64 ± 6.45
p value	0.130	0.038	0.379	0.020	0.018
Marital status					
Single	43.37 ± 6.01	49.60 ± 7.67	60.91 ± 14.99	48.07 ± 6.21	50.49 ± 6.16
Married	38.71 ± 6.56	45.38 ± 8.38	60.41 ± 13.93	43.17 ± 8.23	46.91 ± 5.83
*p*-value	0.004	0.036	0.893	0.005	0.024
Education level					
Illiterate	38.39 ± 7.09	45.14 ± 8.69	59.72 ± 15.26	43.68 ± 8.56	46.73 ± 6.37
Primary school	39.47 ± 6.24	48.68 ± 6.95	61.62 ± 12.69	43.75 ± 7.14	48.38 ± 5.41
Secondary school	40.93 ± 4.97	46.15 ± 9.69	56.41 ± 14.09	42.55 ± 7.28	46.51 ± 5.58
Tertiary	43.33 ± 6.31	47.22 ± 7.82	65.27 ± 11.42	47.92 ± 7.81	50.94 ± 5.42
*p*-value	0.017	0.799	0.677	0.386	0.543
Occupation					
Labor	38.54 ± 6.57	45.09 ± 7.64	59.54 ± 14.16	43.20 ± 8.19	46.59 ± 5.82
Housewife	40.24 ± 6.68	47.22 ± 10.76	61.94 ± 13.35	43.54 ± 8.05	48.24 ± 5.75
Others	43.45 ± 6.03	49.77 ± 8.02	62.73 ± 14.88	48.26 ± 6.23	51.05 ± 6.10
*p*-value	0.021	0.102	0.644	0.058	0.019
Locality					
Urban	44.94 ± 5.38	47.22 ± 7.18	58.68 ± 11.43	47.66 ± 8.01	49.62 ± 5.66
Rural	38.98 ± 6.56	46.18 ± 8.56	60.79 ± 14.47	43.71 ± 8.00	47.42 ± 6.09
*p*-value	0.003	0.655	0.630	0.133	0.230
Duration (n)					
< 2 month (32)	41.24 ± 6.77	46.77 ± 8.24	59.54 ± 12.13	45.06 ± 8.72	48.16 ± 5.99
2–3 months (38)	40.41 ± 6.33	47.26 ± 8.30	63.38 ± 13.74	45.23 ± 7.18	49.07 ± 5.70
> 3 months (25)	36.95 ± 6.54	44.39 ± 8.66	57.53 ± 16.37	41.71 ± 8.24	45.15 ± 6.06
*p*-value	0.038	0.382	0.239	0.178	0.032

The proportions were compared using Independent samples t-test and one-way ANOVA

*p*-value< 0.05 indicating a statistically significant difference

## Discussion

The World Health Organization (WHO), in its constitution, has defined health as “A state of complete physical, mental, and social well-being not merely the absence of disease or infirmity”. HRQOL studies are suitable for understanding the effect of different therapeutic interventions on physical, social and emotional wellbeing of the patients suffering with chronic diseases.

The WHOQOL-BREF questionnaire developed by World Health Organization, a short form of WHOQOL-100, is a cross-cultural instrument. This instrument focused broadly on all aspects of QOL including physical health, psychological, social relationship and environment [25]. As WHOQOL-BREF does not impose a great burden on the respondent it is seen as the most useful instrument to assess QOL [25]. The results of our study have confirmed that WHOQOL-BREF is a reliable instrument to measure QOL among VL patients. This questionnaire is one of the best known instruments that has been developed for cross-cultural comparisons of QOL and is available in many languages. This instrument, by focusing on individuals’ own views of their well-being, provides a new perspective on life. WHOQOL group Instruments were found to be the best tools as far as simplicity and scope of response is concerned.

The development process and ideational of the WHOQOL-BREF scale is stronger than other scales such as SF-36 and the Duke Health Profile. There is a variety of evidence related to the psychometric properties of the scales, and generally there are fewer published studies regarding the Duke Health Profile. The SF-36 has some evidence of responsiveness but might suffer from floor and ceiling effects, while the WHOQOL-BREF does not appear to have floor or ceiling effects. The WHOQOL-BREF has the highest proportion of items that are individualized [26].

In the present study, all domains of QOL among VL patients before receiving treatment was found to be significantly low as compared to healthy volunteers. However, after treatment QOL was drastically improved. The lowest score was found in the physical domain that concerned with physical pain, dependence of medication, energy, mobility, sleep and rest, daily living activities, and working capacity.

Our results seem to be consistent with the findings of other neglected tropical diseases in relation to QOL. Damme-Ostapowicz et al. [20] reported highest score in social domain and the lowest in the physical domain on malaria patients. No significant difference in any domain of QOL was observed among the male and female patients. A similar observation was also reported in patients with post kala-azar dermal leishmaniasis [27] as well as in cutaneous leishmaniasis [17]. Younger patients found to have significantly (*p* < 0.05) better QOL than older age groups. The reason behind this observation could be due to the fact that the older individuals might have lower tolerance to the disease than younger. Another possible reason may be due to physical functioning deteriorates as a result of age related co-morbidities or itself due to aging process [28]. Apart from physical domain, no significant difference was observed in QOL based on level of education. QOL of well-educated people found to be better than illiterate people. The reason may be that people with higher education level are more concern about their health and might have more knowledge towards the disease. This finding is very similar to the findings of another study among HIV patients’ the less educated had a lower quality of life [29]. Patients of VL without formal education were found to have lower QOL this might be due to less awareness of VL. Therefore, education of VL patient’s and awareness program could be a key strategy to prevent severity of the disease. A statistically significant association (*p* < 0.05) regarding marital status was noted. QOL of unmarried patients was better in comparison to married patients. We also got a statistically significant difference with locality, and most affected domains were physical and followed by environmental domains of QOL.

Patients belonging from rural areas found to have lower QOL compare to urban residents, the possible reason of this differentiation could be rural patient may not be having proper diagnosis and treatment facilities near to their houses. As a results, they might not been diagnosed at the initial stage of the disease and progressively the disease become more severe, which in turn may hamper the QOL. Severity was found to be associated with the impairment of QOL in many diseases [27, 30].

As it is already well reported that, Leishmaniasis, especially the visceral form, tends to affect the poorest people [31, 32]. Presence of domestic animals in proximity of the houses with dumping yards which is common in rural areas has been well reported as a risk factor for leishmaniasis this may affect on QOL [33]. A significant correlation was observed in between physical domain and duration of illness. The possible reason behind this could be that, most of the affected patients are poorest of poor belongs to a rural community, and they do not have access to the health care system, which leads to delay in diagnosis and treatment. There were few limitations in our study. First, in this study, we used only one tool (WHOQOL-BREF) to measure the QOL. Second, severity/stage of the disease was not considered. Third, population size of this study was small. Lastly, the prevalence of VL and its associated medical complications is insufficient to explore its impact on the physical, mental, and social well-being of affected individuals.

## Conclusion

The present study has shown that all domains of QOL with VL patients were highly impaired than the control group. Physical and environmental domains were significantly most affected domains as correlated with socio-demographic variables. Diagnosis and treatment of VL is of outmost public health concern in Indian sub-continent particularly in Bihar region. As an outcome measure in future clinical studies, the possibility of using the WHOQOL-BREF instrument for VL patients need to be provoked.

## Abbreviations

SAG: Sodium Antimony Gluconate
VL: Visceral leishmaniasis
WHOQOL-BREF: World Health organisation Quality of Life short form
